# The moral obligation to be vaccinated: utilitarianism, contractualism, and collective easy rescue

**DOI:** 10.1007/s11019-018-9829-y

**Published:** 2018-02-10

**Authors:** Alberto Giubilini, Thomas Douglas, Julian Savulescu

**Affiliations:** 1 Oxford Martin School and Wellcome Centre for Ethics and Humanities, University of Oxford, Oxford, UK; 2 Oxford Uehiro Centre for Practical Ethics, University of Oxford, Littlegate House, St Ebbes St, Oxford, OX1 1PT UK

**Keywords:** Vaccination, Easy rescue, Herd immunity, Moral responsibility, Collective responsibility

## Abstract

We argue that individuals who have access to vaccines and for whom vaccination is not medically contraindicated have a moral obligation to contribute to the realisation of herd immunity by being vaccinated. Contrary to what some have claimed, we argue that this individual moral obligation exists in spite of the fact that each individual vaccination does not significantly affect vaccination coverage rates and therefore does not significantly contribute to herd immunity. Establishing the existence of a moral obligation to be vaccinated (both for adults and for children) despite the negligible contribution each vaccination can make to the realisation of herd immunity is important because such moral obligation would strengthen the justification for coercive vaccination policies. We show that two types of arguments—namely a utilitarian argument based on Parfit’s Principle of Group Beneficence and a contractualist argument—can ground an individual moral obligation to be vaccinated, in spite of the imperceptible contribution that any single vaccination makes to vaccine coverage rates. We add a further argument for a moral obligation to be vaccinated that does not require embracing problematic comprehensive moral theories such as utilitarianism or contractualism. The argument is based on a “duty of easy rescue” applied to collectives, which grounds a collective moral obligation to realise herd immunity, and on a principle of fairness in the distribution of the burdens that must be borne to realise herd immunity.

## Introduction

Despite the success of vaccines in preventing and sometimes eradicating infectious diseases, and despite their demonstrated safety (Navin 2015, p. 6; CDC 2015a; Andre et al. 2008), many people today refuse vaccination for themselves or their children. In the U.S. there has been a significant increase in cases of measles over the last few years due to increasingly widespread non-vaccination: in 2014, for example, there were 667 reported cases, the highest number since measles elimination was documented in the U.S. in 2000 (CDC 2016a). Similarly, in different parts of Europe there were measles outbreaks in 2016 and 2017, due to a significant decrease in measles vaccination rates; for example, in Italy there were more than 3300 cases of measles in the first half of 2017, 88% of which were not vaccinated and 7% of which received just one dose of vaccine (ECDC 2017). Before the introduction of the measles vaccination program in 1963, 3–4 million people in the US were infected by measles every year, and 4–500 of them died (CDC 2015b).

Some reasons for vaccine refusal derive from scepticism about the efficacy or safety of vaccines (Smith et al. 2011; Harmsen et al. 2013), while in other cases objections are based on philosophical or religious views about how humans should deal with diseases; for instance, the largest local outbreak of measles in the US in recent years (383 cases) occurred in 2014 in unvaccinated Amish communities in Ohio (CDC 2016a).

A high rate of vaccine refusal can compromise herd immunity, which is achieved when a sufficient proportion of the population is immune (Andre et al. 2008), and therefore the incidence of infection declines, which makes disease more unlikely to spread (Fine et al. 2011; Dawson 2007). The coverage rate required to realise herd immunity depends on the specific disease considered, but it typically ranges between 90 and 95%.

Herd immunity is a *collective good*, in the sense that it can be produced only through the cooperation of a large number of people (Dawson 2007, pp. 167–168). But herd immunity is also a *public good* (Dawson 2007), in the technical sense of the term: it is non-excludable and non-rivalrous. Herd immunity is non-excludable in the sense that it is not possible to exclude someone from benefitting from herd immunity, even if she does not contribute to the good through vaccination; in fact, everybody benefits from herd immunity, even those who are in any case protected against the disease in question due to their vaccination status, because in a society where herd immunity is realised fewer resources need to be directed to care for the sick. More importantly, herd immunity reduces the risk of infection for (1) those who are too young to be safely vaccinated [e.g. the injectable influenza vaccine is not recommended for children younger than 6 months old, (CDC 2016b)]; (2) those who cannot be vaccinated for medical reasons (for example because they are allergic to certain vaccines or are immunosuppressed); and (3) those for whom vaccination is ineffective [for example, the pertussis vaccine is only 70% effective during the first year and only 30–40% effective after 4 years (CDC 2016c)]. Herd immunity is also non-rivalrous, in the sense that anyone benefitting from it does not reduce the extent to which others can benefit as well.

There has recently been considerable discussion (Pierik 2016; Flanigan 2014; Dawson 2011; Luyten et al. 2011; Verweij and Dawson 2004) regarding whether the state should enforce compulsory or mandatoty vaccination in order to realise herd immunity. In this paper we address a different, though related, issue, namely whether *individuals* have a *moral* obligation to be vaccinated or to have one’s children vaccinated, in spite of the fact that any single vaccination does not make a significant difference to vaccination coverage rates. Thus, the question we aim to answer is the following: how can we justify the existence of an individual moral obligation to be vaccinated or to have one’s children vaccinated, if any individual being vaccinated does not significantly affect a community’s capacity to achieve herd immunity and therefore to protect its vulnerable members?

Two observations about the scope of our arguments are in order. First, we take our argument to apply only to the case of vaccinations protecting against communicable diseases that pose significant risks to the health or life of at least some of those infected. These include, for example, vaccines against seasonal influenza, the MMR (measles, mumps, rubella) vaccine, vaccines against pneumococcal and meningococcal infections, the varicella vaccine, and more generally the vaccines against communicable diseases that healthcare systems recommend for children and adults (see e.g. the communicable diseases, and *only* the communicable diseases, listed at CDC 2016d). Our arguments do not apply to vaccines against non communicable infectious diseases, such as tetanus.

Second, our argument for the existence of a collective and of an individual moral obligation to be vaccinated applies both to adults and to children who can be considered moral agents, i.e., who can appreciate and respond to moral reasons. For example, some vaccines, such as vaccines against meningococcal groups A, C, W and Y disease, are usually recommended for 12 year old children, who certainly count as moral agents and are subject to moral obligations; other vaccines, such as the seasonal flu vaccine, are recommended for children and adults of all ages starting from 6 months old, and many of these individuals certainly are moral agents. In the case of young children who cannot be considered moral agents, and therefore who cannot be subject to moral obligations—such as, for example, 3 year old children for whom the MMR vaccine is recommended—our arguments for the existence of an individual moral obligation with regard to vaccination applies to the parents, who are responsible for decisions about their children’s vaccination; in such cases, the individual moral obligation in question is not that of being vaccinated, but that of having one’s children vaccinated. Now, attributing parents a moral obligation to vaccinate their children might be considered problematic because, whatever the source of such moral obligation, parents also have a moral obligation to act in the best interests of their children. And in certain cases, the obligation to act in the best interest of one’s children provides pro-tanto moral reasons for not vaccinating the children.^1^ For example, it might be in the best interest of a healthy child not to be vaccinated against varicella (chickenpox): since varicella is not a serious or dangerous disease for healthy children, it might be better for the children to avoid the risk of side effects of the varicella vaccine, which range from mild rush to serious anaphylactic reactions (around 1 in 1 million vaccinated individuals), even at the cost of being exposed to the risk of getting the disease. Or, one might suggest, it might not be in the best interest of a child to be vaccinated even against more serious diseases, such measles, in those circumstances in which vaccine coverage rate is already very high: the child would be protected anyway through herd immunity without needing to be exposed to the (very small) risks of vaccination (assuming for the sake of argument that the child will always remain within an area in which vaccination coverage rate issufficiently high). In such cases we would have a clash between two *pro-tanto* moral obligations: the moral obligation to vaccinate one’s children in order to protect other people (whose justification will be the topic of this paper) and the moral obligation not to vaccinate them in order to pursue their best interest. Therefore, in order to demonstrate that parents have not only a *pro-tanto*, but an all-things-considered moral obligation to vaccinate their children we would have to demonstrate that the moral obligation to protect others by vaccinating one’s child outweighs the moral obligation to act exclusively in the best interest of the child. While we will not try to argue for this thesis, we are confident that the arguments we are going to provide are strong enough to make it at least plausible to claim that the *pro-tanto* moral obligation to vaccinate one’s children does outweigh the *pro-tanto* moral obligation not to vaccinate them at least in some cases.

It is important to make the question of individual moral obligation to be vaccinated or to have one’s children vaccinated central to discussion of the ethics of vaccination. If people were convinced that there is an individual moral obligation to be vaccinated or to have one’s children vaccinated and fulfilled this obligation, compulsory vaccination would not be necessary (Dawson 2011, pp. 150–151). Moreover, the existence of a moral obligation is relevant to the justifiability of state-sponsored vaccination programmes. As Marcel Verweij noted with regard to the influenza vaccine, “if citizens have a moral duty to accept vaccination because in this way they will protect others (e.g. the elderly and chronically ill) for whom influenza poses a serious risk, this will give support to a general vaccination policy” (Verweij 2005, p. 324). The thought here is that it will be easier to justify state-sponsored vaccination programmes if these merely encourage or require individuals to do what they in any case have a moral obligation to do than if they encourage or require individuals to go beyond their moral obligations. Thus, it is important to provide a solid justification for the existence of an individual moral obligation to be vaccinated or to have one’s children vaccinated which could strengthen the justification for such vaccination programmes.

### From collective to individual responsibility

The behaviour of a certain number of people is necessary and sufficient to realise herd immunity but no one individual’s actions make a significant difference to whether the effect obtains for the group. This suggests that individuals cannot be morally responsible, in the standard kind of way, for producing herd immunity. However, as we will argue in “Easy rescue, collective obligations, and the individual duty to be vaccinated”, it is possible to attribute moral responsibility to groups in respect of herd immunity. In the retrospective sense of “responsibility”, groups can be *blameworthy* for failing to realise herd immunity, and in the prospective sense of responsibility, groups have a moral *obligation* to realise herd immunity.

Attribution of *individual* responsibility—which is the central issue of this article—is more problematic. Because each individual contribution to the realisation of herd immunity is negligible, it seems problematic to argue that any particular individual has the moral obligation to make her contribution to herd immunity by being vaccinated or by vaccinating their children. Let’s consider vaccination for oneself, and let’s examine two possible scenarios, one in which herd immunity is realised, and one in which it is not. In the first case, where herd immunity is realised, some have argued that since the risk that other people become infected is very small, the ground for attribution of an individual obligation to be vaccinated is weak (Dawson 2007, p. 171). But, as others have observed, one’s contribution to vaccine coverage rates also seems virtually irrelevant if herd immunity is not realised: the risk that other people are infected would be high anyway, regardless of whether one vaccinates, and any effect of one’s decision on the risk of contagion is likely to be negligible (Verweij 2005, p. 329).

Contrary to what these positions maintain, in this paper we argue that individuals do have a moral obligation to be vaccinated or to vaccinate their children even if their contribution to herd immunity is negligible. For brevity’s sake, we will be referring from now on only to the individual moral obligation to be vaccinated, but it is understood that the same arguments apply to the moral obligation to vaccinate one’s children. In “The utilitarian approach: group beneficence and imperceptible contributions” we analyse a utilitarian ethical approach based on Derek Parfit’s Principle of ‘Group Beneficence’; in “The deontological approach”, we consider two possible versions of a deontological approach. We argue that both a utilitarian and a deontological contractualist approach do provide support to the idea that individuals have a moral obligation to be vaccinated. In “Duty of easy rescue and fairness: a further argument for an individual moral obligation to be vaccinated”, we propose a further ethical approach that can justify attribution of individual responsibility to be vaccinated, and that does not presuppose any contentious comprehensive moral theory: we argue that individuals have a moral obligation to contribute to herd immunity by being vaccinated on the basis of a collective duty of easy rescue and of a principle of fairness in the distribution of the burdens that such collective duty entails.

## The utilitarian approach: group beneficence and imperceptible contributions

In *Reasons and Persons*, Derek Parfit discusses the following example:

“a large number of wounded men lie out in the desert, suffering from intense thirst. We are an equally large number of altruists, each of whom has a pint of water. We could pour these pints into a water-cart. This would be driven into the desert, and our water would be shared equally between all these many wounded men. By adding his pint, each of us would enable each wounded man to drink slightly more water - perhaps only an extra drop. Even to a very thirsty man, each of these extra drops would be a very small benefit. The effect on each man might even be imperceptible” (Parfit 1984, p. 76).

The contribution of an extra drop of water to alleviating the men’s thirst may be as imperceptible as the contribution that each individual would make to vaccination rates and to the realisation of herd immunity by being vaccinated. In spite of this imperceptibility, we might still say that each individual has an obligation to be vaccinated in virtue of the same principle whereby Parfit, in his example, attributes to each altruist a moral obligation to make their (imperceptible) contribution to alleviating the wounded men’s thirst. The principle is the following:

When (1) the best outcome would be the one in which people are benefited most, and (2) each of the members of some group could act in a certain way, and (3) they would benefit people if *enough* of them act in this way, and (4) they would benefit people *most* if they *all* act in this way, and (5) each of them both knows these facts and believes that enough of them will act in this way, then (6) each of them ought to act in this way (Parfit 1984, p. 77).

We might call this the Principle of Group Beneficence (Otsuka 1991): each individual member of the collective has a moral obligation to make her contribution to enable the collective to cause the desirable collective effect. The moral obligation of each individual is derived from a principle of utility maximization, since, as per condition (1), the “best” outcome is the one where people are benefited most, and according to Parfit, individuals ought collectively to do what brings about the best outcome. Parfit appeals to an intuition according to which “it is clear” that “each of us should pour his pint into the water-cart” (Parfit 1984, p. 77). If this intuition is veridical, then, given the analogy with the case of vaccination, we should also say that each individual should be vaccinated so as to make one’s contribution to herd immunity.

However, one might object that condition (4) creates a problem for any attempt to apply the Principle of Group Beneficence to cases of imperceptible individual contributions to a collective effect. According to that condition, the moral obligation to make one’s small contribution to a desirable collective effect exists where a group of individuals would produce the most benefit if *all* the individuals acted in a certain way. The principle might in itself be valid. However, at a first glance it seems to apply neither to Parfit’s example nor to the case of individuals’ contribution to herd immunity. Recall that in Parfit’s example, and also in the case of vaccination, each individual’s contribution to the collective effect is imperceptible. Accordingly, it would seem that if all but one of the people (and therefore *not all* individuals) poured their pint into the water-cart, the benefit to the thirsty men would not be significantly smaller than the benefit they would get if all poured their pint. Suppose one of the altruists is also thirsty: it seems that utility would be maximized if this individual drank her pint of water instead of pouring it into the water-cart, because her thirst would be alleviated at no significant cost to the wounded men. In the same way, one might say, if all but one person were vaccinated, vaccination coverage rates would not be significantly lower than they would be if all were vaccinated, since each individual contribution to coverage rates is negligible. Suppose a person is opposed to vaccination: utility would be maximized if this individual was not vaccinated, because she would have her anti-vax preference satisfied without any significant impact on coverage rates or herd immunity. For the same reason, the situation in which all but two people are vaccinated is better, from the point of view of utility maximization, than the one in which all but one are vaccinated; and so forth.

The same reasoning might be iterated also beyond the point at which the non-vaccinated people are so many that herd immunity is lost. In those cases, herd immunity would not be realised anyway, even if one more individual contributed by being vaccinated. In such cases, one might be tempted to agree with Marcel Verweij that “if most people forgo vaccination against influenza, the effects on public health of my choice for vaccination become negligible” (Verweij 2005, p. 329). Therefore, as far as utility maximization is concerned, “if non compliance is common, my obligation to contribute to prevention will weaken or even fade away” (Verweij 2005, p. 330).

Thus, whether or not herd immunity is realised, any individual might be able to claim that the imperceptibility of her contribution implies that she does not have the moral obligation to be vaccinated.

However, there is more to say in support of Parfit’s intuition, and in support of the idea that condition (4)—namely that individuals would benefit people *most* if they *all* contributed through pouring their pint of water in the water tank (or through being vaccinated)—does apply to the case of realisation of herd immunity. An analogy with a relevantly similar case might provide support to Parfit’s claim that “each of us should pour his pint into the water-cart” because people would benefit most if all contributed. Parfit’s example discussed above is relevantly similar to one in which there is the same amount of water, the same number of potential water-givers, and the same number of water-recipients, but each water-giver’s pint, instead of being collected into a water-cart, goes directly to a single thirsty man, and thus makes a significant difference to a single person (for an analogous case, see Glover 1975, pp. 174–175). On this variant of the case, if one person does not pour her pint, then one thirsty man gets no water and dies. In such cases, it seems obvious that each man ought to donate her pint of water. But it does not seem that it should make a moral difference whether exactly the same donation is directed to a specific individual (call this “directed donation”) or is collected together with other donations and then distributed among all the thirsty individuals (call this “collected donation”). It seems at least plausible to suggest that the difference in the method of delivery of water is morally irrelevant. Therefore, one might continue, if it is wrong not to make your directed donation, and considering that the difference between the directed and the collected donation is morally irrelevant, it is wrong not to make your collected donation either, i.e. not to pour your pint into the water-cart.

Now, one might reply by questioning the moral equivalence between directed and collected types of donation in Parfit’s example—after all, one might say, it does make a moral difference, from a consequentialist perpective, whether one’s pint of water is given to a specific individual or simply collected in a water cart: a single donation has perceptible consequences in the former but not in the latter case. However, the moral equivalence between directed and collected types of benefit seems less controversial in the case of individual contributions to herd immunity. Although each person who is vaccinated contributes only imperceptibly to vaccination rates—and therefore vaccination is in this respect analogous to collected donation—, vaccination might be decisive in whether or not another person is infected—and therefore vaccination is in this respect analogous to directed donation. Each person who is not vaccinated, although contributing only imperceptibly to herd immunity, might be the one who infects another, in the same way as each person who does not contribute her pint of water is paired with a thirsty person who does not receive any water in the case of directed donation. In the case of vaccination, then, directed and collected types of benefit overlap. Suppose that each individual non-vaccination constitutes a risk of infecting others of, say, 0.1%. This means that, statistically, one in every 1000 non-vaccinated individuals will infect another person. But anyone can be the non-vaccinated person who infects others. When infection happens, not being vaccinated is to be considered as analogous to not giving one’s pint of water in ‘directed donation’ for the purpose of attribution of moral responsibility: in both cases a person suffers or might even die for an easily preventable cause, and in both cases there is one person who could have prevented the harm and who is paired with the person who suffers the consequences of the first person’s choice. Accordingly, we can conclude that, given the risk of infecting others posed by any non-vaccinated person, given that the cost to each individual of avoiding this risk is small (considering the safety of vaccines), and given that infection can be an extremely negative outcome (which in some cases can cause the death of the infected individual), a utilitarian approach does justify a moral obligation to be vaccinated in spite of the imperceptible contribution of each vaccination to vaccine coverage rates.

The fact that there is only a small risk that infection will occur does not mean that individuals do not have a moral obligation to avoid the risk of being the ones who infect others: to the extent that the expected harm to others of non-vaccination remains larger than the expected harm of vaccination to the vaccinated individual (which seems to be the case, considering the proven safety of vaccines), the expected utility of non-vaccination is negative, and therefore utilitarianism implies that there is a *prima facie* moral obligation to be vaccinated. In fact, the only significant difference between the case of directed water donation and the case of vaccination is that in the former case the harm inflicted on other persons if individuals fail to make their contribution is certain, whereas in the latter case there is only a risk of inflicting harm on any individual. However, this difference is not significant enough to render non-vaccination permissible from a utilitarian point of view. If the harm of infection is large, even a small risk of causing infection due to non-vaccination may be sufficient to generate a significant expected harm.

As Dawson has noted, every single vaccination matters morally, at least where herd immunity does not exist, because without herd immunity any individual non-vaccination increases the risk of a significant harm to others “even if in an infinitely small way” (Dawson 2007, p. 170). Therefore, Dawson claims, “where we can perform an action to reduce the risk of foreseen harm to others through undergoing vaccination (at least where herd protection does not exist), then we may be obligated to do so” (Dawson 2007, p. 171). The analogy with directed water donation provides support for this claim, but also allows us to extend the same consideration to cases in which herd immunity does exist. After all, where the risk is of a very bad outcome and is applied to a large population, the fact that non-vaccination only very slightly increases the risk of infecting others (such as in cases where herd immunity does exist) does not make such risk insignificant.

Thus, a utilitarian approach supports a moral obligation to be vaccinated, *unless the individual cost of being vaccinated would be so great as to outweigh the expected negative contribution of non-vaccination to the aggregate wellbeing of others*. To compare, we can think of a case in which each of the men with the pint of water is also extremely thirsty to the point that they are about to die, and at least as thirsty as any of the wounded men in Parfit’s example. In such case, utilitarianism would not ground a moral obligation for him to give away her pint of water to relieve another person’s thirst. However, as a matter of fact, vaccination does not pose any significant cost to most individuals, and actually it provides significant benefits. Vaccines are very safe and effective, the risks of side-effects or iatrogenic diseases is very small, and there are typically also significant benefits in terms of protection from life-threatening diseases for the vaccinated individual (Andre et al. 2008). For example, the MMR vaccine is very safe when administered to healthy adults who are not alergic to the vaccine: the most common side effects consist at most in pain at the injection site, fever, a mild rash, and temporary pain and stiffness in the joints; the most serious side effect, immune thrombocytopenic purpura (ITP) (a disorder that decreases the body’s ability to stop bleeding), has been observed in children, not in adults, and is in any case extremely rare, amounting to 1 case every 40,000 vaccinated children (CDC 2015a). In contrast, mumps, measles, and rubella can have serious and potentially fatal complications, including meningitis, swelling of the brain (encephalitis) and deafness; measles’ fatality rate is around 0.2% and (CDC 2016e) and acute encephalitis occurs in approximately 0.1% of reported cases; rubella in pregnancy can also result in serious birth defects and miscarriages (NHS 2015).

We can therefore refine the analogy with the case of water donation so as to reflect the fact that the cost to each individual of being vaccinated is small. We can compare the case of vaccination with a case in which 1000 people have 5 Ls (instead of 1 L) of water each, and each of 1000 thisty men needs only 1 L to alleviate their thirst. In this case, the cost to each individual of pouring 1 L in the water tank or of giving 1 L of water to a specific thirsty man is very small, because each individual can keep 4 Ls for herself. This analogy seems to better reflect the small cost that vaccination poses on each individual.

Thus, in spite of the imperceptible contribution each single vaccination makes to vaccine coverage rates and therefore to herd immunity, a utilitarian approach to vaccination does justify a moral obligation to be vaccinated, at least in cases in which the expected utility of non-vaccination for others is negative (there is a small risk of an extremely bad outcome), and the cost to each individual of preventing the negative outcome is small (we will discuss below in “High cost vaccinations” the case of individuals to whom the cost of vaccination is not small).

While the utilitarian justification for a moral obligation to be vaccinated applies to anyone (except those for whom vaccination would involve high risks), it is particularly compelling for people who are more likely to be exposed to infectious diseases and to infect others, such as health workers.

## The deontological approach

This section explores two deontological approaches to responsibility for herd immunity: a (non-Kantian) approach based on universalization, or the generalization of a maxim of non-vaccination, and a contractualist theory.

### Vaccination and the generalization test

The first deontological approach is based on a particular understanding of the universalizability test, which is often considered as the decisive test for assessing the morality of certain behaviours. According to this view—which is also known as the “generalization test” (Glover 1975, pp. 175–176)—a certain action is wrong if the consequences of everybody acting in that way would be significantly bad, even if the consequences of any one particular person acting in that way are not bad. In other words, according to this account, the question we should ask in order to assess the morality of a certain action is: what if everybody acted in this way? And in particular: what if everybody refused vaccination (for themselves or for their children)? Clearly, universal non-vaccination would have very bad consequences. So one might say that this moral approach renders non-vaccination immoral even if the consequences of any one person not being vaccinated are not bad. There are however two objections—at least one of which is decisive—that can be raised against the generalization test as a valid criterion for moral assessment.

First, one might appeal to the same (act) consequentialist objection that Shelly Kagan offered against the generalization test. The objection is that the generalization test is “in tension with the thought that the rightness or wrongness of a given act should depend upon the consequences of that act” (Kagan 2011, p. 112), rather than on counterfactual considerations about the consequences of hypothetical agents all acting in the same way. If we agree with Kagan’s objection, we have to conclude that the generalization test does not represent a reliable method of moral assessment, and therefore that the question “what if everybody refused vaccination?” is irrelevant for a moral assessment of non-vaccination. This objection to the generalization test is however only available to those who subscribe to act consequentialism.

A second—and decisive—objection to the generalization test is based on the idea that, for the purpose of applying the generalization test, a full description of the action in question should include the description of all the circumstances which characterize that action (Glover 1975, pp. 176−77). But refining further the description of the action to be generalized would undermine the generalization test, because “the more complete in the relevant respects the description becomes, the closer the generalization test comes to giving the same answer that one gets to the question “what will happen if *I* do this?” (Glover 1975, p. 176). Thus, for example, at least in cases where herd immunity is realised, a person who chooses not to be vaccinated because she *knows* that, say, 95% of other people will be vaccinated anyway, could claim that her action is to be described not simply as ‘non-vaccination’—whose generalization would have bad consequences—but more specifically as ‘non-vaccination on the basis of the knowledge that enough other people around me are vaccinated’. Thus, the answer to the question ‘what would happen if everybody were not vaccinated in the circumstances I find myself in?’ would be equivalent to the answer to the question ‘what would happen if I were not vaccinated?’. The generalization test boils down to an act-consequentialist assessment of non-vaccination. Therefore, the generalization test would be redundant: we do not need it to decide the morality of non-vaccination, which would ultimately depend on whether or not we accept act-consequentialism.

### Vaccination and contractualism

A second possible deontological approach is contractualism (see Ashford and Mulgan 2012). According to contractualism, people should act upon principles to which all could accede, or would accede under some specified ideal circumstances. Scanlon’s (1998) classic formulation holds, more precisely, that

[a]n act is wrong if its performance under the circumstances would be disallowed by any set of principles for the general regulation of behaviour that no one could reasonably reject as a basis for informed, unforced, general agreement. (Scanlon 1998, p. 153).

Marcel Verweij holds that, when applied to the case of vaccination, contractualism is a very demanding theory. As he writes, “[p]ersons most vulnerable to the disease do not respond optimally to vaccination (…) and therefore they will be much better protected if everyone were vaccinated, the old and the young, the ill and the healthy” (Verweij 2005, p. 333). It seems therefore that a person could not justify her decision not to be vaccinated to the vulnerable members of the community who are at risk of contagion, as contractualism would require.

One might reply that the additional risk that any non-vaccinated person would pose to others is negligible, as is the additional contribution of the individual to coverage rates and to herd immunity. Accordingly, it might seem that a person could justify her decision not to be vaccinated to vulnerable people. For example, the principle regulating her behaviour might be something like ‘I won’t be vaccinated if I know that enough other people in my community are vaccinated anyway’, or ‘I won’t be vaccinated if I know that few people in my community are vaccinated anyway’. In either case non-vaccination would pose only a small additional risk to vulnerable people. However, such a small additional risk does make a difference in terms of the moral assessment of non-vaccination not only in a utilitarian perspective (as seen above), but also within a contractualism framework. It is rational for each person who is at risk of infection to demand that others contribute to keeping this risk of infection to a minimum. When keeping the risk of infection to a minimum comes at a small cost to others, as is the case with vaccination, this demand by persons at risk is not only rational, but also reasonable. We are here defining “reasonable” in such a way that the objective costs one has to bear, such as the risks of side-effects the vaccinated takes on herself, make the option in question reasonable or an unreasonable to reject; thus, the small risks involved make a principle that prescribe to be vaccinated, in this sense, unreasonable to reject. However, some would insist that the psychological or emotive costs of vaccination would remain very high for those who have deeply held religious or moral beliefs against vaccination or who are genuinely scared of the possible side-effects of vaccines. We will address the issue of vaccination that are high cost in psychological or emotive terms in “High cost vaccinations”.

Thus, contractualism can ground a moral obligation to be vaccinated, at least as long as vaccination entails a small cost to individuals.

Verweij says that “contractualism requires us to take precautions that seem to be excessive” (Verweij 2005, p. 334), and more generally some have claimed that contractualism is a too demanding ethical theory (Ashford 2003). Whether or not this is true, contractualism does not seem to be too demanding in the case of vaccination, and therefore this objection is not available to those who wish to reject a contractualist justification of a moral duty to be vaccinated. The precautions that contractualism requires us to take do not seem to be excessive in the case of vaccination, considering the very small individual cost that vaccination entails, at least for the vast majority of individuals (again, we will address the issue of the moral obligation of those for whom vaccination would entail a high cost below in “High cost vaccinations”). Thus, regardless of whether contractualism is in itself a too demanding theory, to the extent that vaccination entails a small cost to individuals we can certainly appeal to contractualism to justify a moral obligation to be vaccinated, in spite of the negligible contribution of each vaccination to the realisation of herd immunity.

## Duty of easy rescue and fairness: a further argument for an individual moral obligation to be vaccinated

We have seen that both a utilitarian and a contractualist approach can justify a moral obligation to be vaccinated (or to vaccinate one’s children) in spite of the negligible individual contribution to herd immunity, at least if non-vaccination constitutes a small additional risk of infecting others that can be prevented at a very small cost to individuals. One might reply, however, that appealing to utilitarianism or to contractualism is problematic because it requires accepting comprehensive moral theories to which many reasonable people do not subscribe. Both utilitarianism and contractualism are often considered very demanding theories. In fact, they would justify an obligation to be vaccinated even if the cost to individuals were not small, as long as the benefits to others of vaccination remain sufficiently large, and some may reject the theories on this basis. Thus, in this section we seek to offer a more ecumenical justification for a moral obligation to be vaccinated, one that renders it unnecessary to appeal to utilitarianism, contractualism, or any other contested, comprehensive moral doctrine. Our justification appeals to a duty of easy rescue applied to collectives that could be endorsed by proponents of a wide range of such doctrines.

### Easy rescue, collective obligations, and the individual duty to be vaccinated

A duty of easy rescue is an almost uncontroversial requirement of morality, i.e. a requirement on which most reasonable people would agree, no matter what moral theory or moral view they subscribes to (with the exception, perhaps, of some libertarians). According to the duty of easy rescue, when I could do something that entails a small cost to me and a significant benefit to others, I have a moral duty to do it. Peter Singer provided perhaps the most famous characterization of the duty of easy rescue in his article *Famine, affluence, and morality*, through the well-known example of the child drowning in a pond. The case is analogous to all the cases in which an agent could easily avoid serious harm to someone else without significant personal costs. According to Singer,

“if I am walking past a shallow pond and see a child drowning in it, I ought to wade in and pull the child out. This will mean getting my clothes muddy, but this is insignificant, while the death of the child would presumably be a very bad thing” (Singer 1972, p. 231).

The duty of easy rescue as expressed by Singer’s example does not presuppose, nor does it support (though it is consistent with), a utilitarian morality. A formulation of the duty of easy rescue has been provided by Tim Scanlon, according to whom, “[i]f we can prevent something very bad from happening to someone by making a slight or even moderate sacrifice, it would be wrong not to do so” (Scanlon 1998, p. 224). Thus, both utilitarians (such as Peter Singer) and contractualists (such as Tim Scanlon) have endorsed a duty of easy rescue. This is not surprising: we have seen above that both a utilitarian and a contractualist ethical approach support a moral obligation to be vaccinated and to reduce the small additional risk of infecting others at least *as long as this comes at a small cost to individuals*.

Now, considering the small cost to individuals of vaccination, being vaccinated is comparable to getting one’s clothes muddy in Singer’s example, or to donating one’s litre of water in Parfit’s example (or perhaps, one could plausibly argue, it is even less costly, given its benefits). However, the desirable outcome—namely herd immunity—cannot be realised individually. Herd immunity is a “collective effect”: it requires the contribution of a sufficiently large number of individuals to be realised. Accordingly, if there is a moral duty to realise herd immunity, such moral duty will arguably need to take a collective, rather than an individual form: no individual can realise herd immunity, in the same sense in which no individual can form a circle. Many would take this to imply that no individual can have a duty to realise such immunity, since ‘has a duty to’ implies ‘can’.

As is the case with individuals, one uncontroversial way to justify the collective moral responsibility to realise herd immunity is to say that such responsibility expresses a duty of easy rescue, and more precisely a collective duty of easy rescue: realising herd immunity would be a collective moral obligation if it came at a very small cost to the collective. We can express the principle at the basis of the collective duty of easy rescue in the case of herd immunity as follows:

If a collective could realise herd immunity, then this collective ought to realise herd immunity, provided that the collective cost is small and can distributed in such a way that the cost borne by each individual is also small (so that the collective cost is small under any plausible understanding of “collective” and that the collective duty is consistent with an individual duty of easy rescue)

It is not difficult to see how the principle grounds a collective moral duty to realise herd immunity: the small individual cost of vaccination entails that the cost to the collective of fulfilling its duty is also very small, because it merely consists of the aggregate individual small costs of vaccination, and there is no additional cost that the collective has to bear; at the same time, the benefits of realising herd immunity are very large. Therefore, there is the collective moral duty, grounded in a collective duty of easy rescue, to realise herd immunity. For the sake of consistency with the terminology used in the relevant literature, we will refer to this collective duty also with the terms “collective responsibility” and “collective obligation”: for the purpose of the present discussion, these terms are to be understood as synonymous.

Now, who exactly is the bearer of such responsibility? Is it borne by the collective as a unified agent? Is it somehow distributed across the individuals that comprise the collective? Or both? Here, we need to distinguish two questions: the first is a conceptual question: *what does it mean* to attribute moral responsibility to a loose collection of individuals—understood as a collection without a decisional procedure and an internal structure—like the individuals who together could realise herd immunity? The second question is instead genuinely ethical: what are the *ethical implications* of attribution of collective responsibility to loose collections of individuals for attribution of individual responsibility?

The debate on collective responsibility of loose collectives has focussed mainly on the former question, providing different answers (see e.g. Wringe 2016, 2010; Aas 2015; Pinkert 2014; Björnsson 2014; Collins 2013; Schwenkenbecher 2013; Lawford-Smith 2012; Isaacs 2011). To give just a quick overview of the types of concepts involved, the collective responsibility of loose collection has been conceived as a “joint” responsibility or duty (Pinkert 2014; Schwenkenbecher 2013), or as “shared” responsibility (Björnsson 2014), or as a “putative” collective responsibility (Isaacs 2011 and 2014), or as a form of responsibility attributed to a collective agent (Aas 2015), or as a form of responsibility that “supervenes” on individual responsibilities (Wringe 2016). However, we do not need to go into the details of each of these positions here, and therefore we are not going to provide a definition of each of these concepts. Rather, here we want to address directly the second, ethical question about what the attribution of a collective obligation to a loose collection of individuals implies, for an ethical point of view, in terms of attribution of individual responsibility.

We suggest that an ethical analysis of collective responsibility can allow the derivation from the existence of a collective duty of easy rescue of an individual *duty to contribute* to the relevant collective outcome, which, in the case of a collective obligation to realise herd immunity, translates into an individual duty to be vaccinated (or to vaccinate one’s children). More in particular, the collective obligation to realise herd immunity implies a duty for each individual to contribute to herd immunity—and therefore to be vaccinated—on the basis of a principle of *fairness* in the distribution of the burdens entailed by the collective moral obligation, or, as George Klosko put it, on the basis of a “just distribution of benefits and burdens” (Klosko 2004, p. 34). The burdens consist in people having to pay a visit to the doctor, receive an injection, incur the (very small) risk of side effects of the vaccine and of iatrogenic disease, and, for those who have reservations about the ethics of vaccination, overcome such reservations. A principle of fairness requires that such burdens be distributed fairly across individuals, and therefore that each individual take on herself a fair share of the burdens entailed by the collective obligation by being vaccinated, unless being vaccinated is too burdensome for the individual (we will consider these rare cases in “High cost vaccinations”). This means that the type of collective responsibility that is entailed by the duty of collective easy rescue can be understood in a merely *distributive* sense (Held 1970). We can take the notion of *distributive* collective responsibility to indicate that *all* members of a sufficiently large group have an individual obligation to *contribute* to the realisation of a desirable collective effect, such as herd immunity, and that in virtue of a principle of fairness in the distribution of the burdens entailed by a collective obligation such individual obligation exists in spite of the fact that any individual’s contribution to coverage rates and to the realisation of herd immunity is imperceptible.

Notice that our argument is *not* the same as the argument according to which the non-vaccinated would be impermissibly free-riding on herd immunity (Navin 2013, pp. 70–75, 2015, pp. 143–144; van den Hoven 2012; Dawson 2007, pp. 174–176), although the impermissibility of free-riding is an implication of our argument. To use once again George Klosko’s words, the problem is not so much that “[i]ndividuals who benefit from the cooperative efforts of others have obligations to cooperate as well” (Klosko 2004, p. 34), and therefore that individuals would violate a requirement of reciprocity. Rather, the unfairness implied by the decision not to contribute to herd immunity is, at a more fundamental level, the unfairness of failing to make one’s contribution to fulfilling a collective obligation that is ascribed to the collective of which we are part, i.e. the collective that can realise herd immunity from any disease. The conceptual distinction between the two types of unfairness also has significant practical implications. For example, suppose that in a given community there is herd immunity against measles but not against HPV. In such a context, the unfairness of free-riding on herd immunity implies that a person has a moral obligation to be vaccinated against measles, but not against HPV, given that in the case of HPV there is no herd immunity on which this person can free-ride. However, our argument based on the unfairness of failing to make one’s contribution to a collective good like herd immunity implies that a person has a moral obligation to be vaccinated against both, given that herd immunity against both measles and HPV is a valuable societal goal that that community has a moral obligation to realise or to preserve. Or consider the following:^2^ sometimes, in polio outbreaks, healthy people who have been already vaccinated with the inactivated polio virus (IPV) are called to take the oral polio vaccine (OPV) as well in order to disseminate it in the benefit of the vulnerable: the attenuated vaccine virus in the OPV replicates in the intestine and is then excreted, which allows it to spread in the immediate community (WHO 2017). Thus, those taking OPV do not need vaccination for themselves at all if they have already been vaccinated with IPV; they only take OPV in order to benefit their community. Once again, while an argument based on the unfairness of free-riding would not imply that these healthy people have a moral obligation to take the OPV—because, being already vaccinated, they would not “benefit” from herd immunity (except in the broad sense in which everyone benefits from living in a society without polio)—our argument based on a fairness based obligation to make a contribution to a public good does imply that they have such a moral obligation, though it may be a weaker one to the extent that the benefit of the vaccination is smaller in such cases.

Also, our argument can be taken to have some implications for the issue of whether, in case of an infectious disease outbreak, those who refused vaccination for themselves or for their children for non medical reasons should be held accountable, or morally responsible, for the whole outbreak, i.e. even if they have not directly infected anyone or only infected a few people. The answer to this question depends on whether the notion of ‘accountability’ or ‘moral responsibility’ we are using presupposes causal responsibility. If we think that accountability or moral responsibility presuppose causal responsibility, i.e., that someone can be accountable, or morally responsible, for outcome x only if they are to some extent causally responsible for x, then of course a non-vaccinated individual is not accountable for the outbreak unless she infects so many people that the number of people infected by her is so great that it constitutes by itself an outbreak (which is unlikely), or unless the chain of infections that spread among the population can all be traced back to her as the one who started the contagion. However, if we think that accountability or moral responsibility do not presuppose causal responsibility, i.e. that someone can be held accountable, or morally responsible, for x even if she does not play any causal role in bringing about x, then our argument does imply that she is accountable, or morally responsible, for the outbreak, because she failed to fulfil her moral duty to make her fair contribution to the prevention of the contagion, regardless of whether her contribution would have made a difference. In the same way we can say, for example, that I am accountable or morally responsible for global warming if I engage in practices that involve unnecessary release of carbon emissions, even if the quantity of my carbon emissions does not make any difference to whether global warming occurs.

Let us address two possible objections to our arguments.

The first objection is that individuals have a right to bodily integrity, which includes a right not to have any external substance injected in their body. This right to bodily integrity could be thought to outweigh any moral obligation to be vaccinated. In this view, making one’s fair contribution to herd immunity would be morally different from making one’s contribution to any other collective or public good that does not involve the violation of individual rights to bodily integrity. A right to bodily integrity can be understood in either of two senses: either as a right not to undergo any invasive medical procedure without one’s consent or as a right not to have external substances introduced in one’s body without one’s consent. However, on either of these two understandings, this objection misconstrues the nature of the right to bodily integrity. The right to bodily integrity is normally understood—with reference to Hohfeld’s analysis of rights—as a claim-right held against others. It is a right that others not interfere with one’s body in certain ways, and perhaps also that they provide one with certain forms of assistance required for the minimal functioning of one’s body. Understood thus, the right to bodily integrity may imply that others are under a *pro-tanto* duty not to impose vaccination on an individual without their consent. However, the claim-right to bodily integrity held *against others* does not imply that one is under no moral duty to vaccinate oneself: having a claim right to non-x (e.g., to non-vaccination) is quite consistent with having a moral duty to x. In other words, if you have a right to bodily integrity, the right will entail that other people are under a duty not to force the vaccine on you. But it does not imply anything about what you morally ought to do. Thus, in principle, an appeal to the right to bodily integrity does not constitute a reason against the existence of a moral duty to be vaccinated. Of course, in practice, when vaccination is imposed by parents or medical professionals on children, children may possess a right to bodily integrity that is infringed by this imposition. However, it should be noted that, if the child is competent to consent, their valid consent could be obtained prior to vaccination, and this would prevent the vaccination from infringing the right to bodily integrity. In that case, the parents could still have an obligation to vaccinate their child, conditional on the child consenting to this. If consent cannot be obtained, then whether the pro-tanto duty not to impose vaccination that derives from a right to bodily integrity represents an all-things-considered duty not to vaccinate a child without their consent will depend on (I) how weighty the right is relative to the goods that can be achieved by forcible vaccination and the values that can be promoted (e.g., fairness), and (II) whether imposing vaccination involves bodily interference of the sort that infringes the right to bodily integrity, which remains to be established. Thus, parents might still be under a moral obligation to vaccinate their children even if this entails some violation of their right to bodily autonomy. In any case, even if the right to bodily autonomy is very weighty, it does not represent an argument against a duty to be vaccinated or to vaccinate one’s children, but only an argument against *the enforcement* of such duty.

The second objection is that, as Pinkert put it in his criticism of Wringe’s distributive notion of collective responsibility, moral obligations to *contribute* to a collective outcome, without any further qualification, “imply that you ought to contribute even if not enough others contribute as well”; however, Pinkert continues, “it is implausible that one ought to perform such pointless actions” (Pinkert 2014, p. 189). Admittedly, our distributive notion of collective responsibility grounded in a principle of fairness implies that any individual has a moral obligation to contribute to herd immunity regardless of whether other members of the collective do their part, and therefore even if her contribution is pointless. Requiring everybody to be vaccinated regardless of how many people around them are vaccinated might sound implausible because a principle of utility conflicts with a principle of fairness: fairness would require to choose an option, namely vaccination, that has no utility net, and actually has a (small) cost for the individual. Our reply to this objection is twofold.

First, even if the act of vaccination is “pointless” as a contribution to herd immunity, it is not pointless on a utilitarian assessment. As we saw in the section on utilitarianism, it is simply not true that the utility net of any individual vaccination would be zero, given that any non-vaccinated person has a small chance of infecting others. Therefore, in this perspective, each individual vaccination is not pointless: vaccination might not be morally obligatory as a “contribution” to herd immunity, but it would still be morally required in order to minimize the risk of infecting others. In this sense, vaccination is different from the contribution to any other outcome that requires the imperceptible contribution of each of a large number of individuals, such as filling the water cart in Parfit’s example. There is a small chance that any vaccination would make a difference not qua “contribution” to herd immunity but in terms of infection prevention. Therefore, fairness does not conflict with expected utility: they both imply that individuals ought to be vaccinated. Moreover, another consideration in support of the idea that individual vaccination has positive expected utility and therefore that the moral obligation to be vaccinated can be supported on utilitarian grounds is that community efforts to cultivate herd immunity are often projects only of domestic politics; however, the fact that people in the globalized world travel at an unprecedented rate implies that exposure to vaccine-preventable diseases is only a plane trip away. Therefore, individual vaccination provides expected net utility given that, even when there is herd immunity, there still is vulnerability for individuals in the global context.

Second, fairness would still demand that any individual contributed to herd immunity even if the contribution, *qua* contribution to herd immunity, would be pointless. There are two possible scenarios here: either the individual contribution, *qua* contribution to herd immunity, is pointless and herd immunity exists, or the individual contribution, *qua* contribution to herd immunity, is pointless and herd immunity does not exist. In the first case, every individual would be under a moral obligation to be vaccinated to ensure that those who are vaccinated are not unfairly burdened, even if any contribution, *qua* contribution to herd immunity, is pointless. Compare the case of vaccination with the case of taxation: society would probably be able to tolerate a certain number of free-riders, who enjoy but do not contribute through their taxes to the maintainance of certain public goods; actually, overall utility would probably be maximized if a few individuals were allowed not to pay taxes, because, without their money, certain public goods, e.g., national defense or a good health care system, could still be guaranteed, and they would get to save some money. However, we are not inclined to tolerate tax evasion regardless of the impact that tax evasion has on the capacity of a state to provide certain public goods, because, at least where the vast majority of individuals do pay their own taxes, we expect all individuals to make their fair contribution: if I am under a moral or a legal obligation to pay my fair amount of taxes, we expect that everybody else is or should be, even if this would not maximize utility. The same considerations can be applied to the case of individuals’ fair contribution to the public good of herd immunity.

Consider now the second possible scenario—where any contribution qua contribution is pointless and herd immunity does not exist. Admittedly, in this case it is more difficult to argue that everybody has a fairness based moral obligation to be vaccinated. As Navin put it, “I have a duty of fairness to contribute to herd immunity only if most other members of my community act on the basis of this duty. If they do not, then herd immunity will not exist, and, therefore, I will not have a duty of fairness to contribute to it” (Navin 2015, p. 180). There are two possible replies here, the first of which is probably stronger than the second.

The first reply is that, as we have said above, in such cases considerations of fairness would be replaced by welfarist considerations or considerations of general beneficence: especially where vaccination rate is low, any non-vaccinated individual would significantly increase the risk that others be infected, so any individual would still have a duty to be vaccinated in order to minimize the risk of harming others.

Second, it is not so obvious that where too few people contribute to a public good and the public good is therefore not realised, there is no duty of fairness to nonetheless make one’s contribution. One could plausibly argue that even if most people around me did not pay their taxes, I would still have a moral duty to pay my fair share, on condition that what is considered fair is not determined by the fact that others do not make their fair contribution—for example, on condition that I am not requested to pay more taxes to compensate for the fact that many people around me do not pay theirs. This condition however does not apply to the case of vaccination, given that there is nothing more that any individual could do to contribute to herd immunity than to be vaccinated (or to vaccinate her children). Therefore, one could plausibly argue that I have a duty of fairness to be vaccinated even if few people around me are vaccinated in the same way as I have a duty of fairness to pay my fair (but no more than my fair) share of taxes even if many people around me do not. Admittedly, though, if such a moral duty existed, it would be quite a weak duty, and indeed it would be the weaker, the higher the number of people around me who fail to make their contribution. Also, the intuition that there is even a weak moral duty to make one’s contribution in such circumstances is probably not widely shared.

### High cost vaccinations

Mark Navin holds that one’s contribution to herd immunity is fair when the cost is not only roughly the same for everyone, but also “reasonable”, i.e. not overdemanding (Navin 2015, p. 142). This restriction seems appropriate in light of a duty of (individual) easy rescue and to the extent that, generally speaking, the cost individuals *normally* have to bear for being vaccinated is small.

But what kind of cost can be considered large enough to outweigh the moral duty to be vaccinated, i.e. to make vaccination supererogatory? One might suppose that it would make a difference whether an individual has health insurance (or is anyway covered by a national healthcare system). Without health insurance, any possible side effect of vaccination could potentially become a great burden. However, we need to consider that, as we said above, side effects of vaccines are very rare, and the most common of these rare side effects are not serious. Thus, the risks of vaccination for the uninsured remain very small, and indeed, though we cannot argue for this point here, we believe they are so small that they are insufficient to undermine the moral obligation to be vaccinated.

Admittedly, though, the cost of vaccination is not always small. Some people are too young or too old to be vaccinated, some people are allergic to vaccines, some people are immunosuppressed. Vaccination would be unsafe for these individuals, and therefore the cost to them of being vaccinated would not be small. Fortunately, our arguments do not imply that individuals have an obligation to undergo *high cost* vaccinations. Indeed, our requirement that the costs of fulfilling the collective duty to realise herd immunity must be distributed fairly could be invoked in support of the view that individuals need not undergo high cost vaccinations. Such high cost vaccinations would be unfair, since these individuals would be required to bear a greater share of the costs of realising herd immunity than others. As noted above, fairness requires that individuals bear similar costs.

Besides, our formulation of the collective duty of easy rescue requires that the costs for all individuals be small. This means that those for whom vaccination would represent a high cost will have to be excluded from the collective that is subject to the collective obligation, in order for the collective to fall under the duty in the first place.

It might be the case that some vaccinations entail high costs of a psychological nature, for example when parents have religion based objections to vaccines that use materials from cell lines derived from aborted foetuses or experience severe psychological distress worrying about potential vaccine complications for their vaccinated child. Perhaps in such cases fairness-based reasons are not strong enough to justify the existence of a duty to vaccinate, given that one might object that vaccinating in such cases is supererogatory. Now, it is not clear whether the moral significance of the psychological costs involved outweighs the strength of fairness-based reasons. But let’s assume, for the sake of argument, that it does. Even if that is the case, the problem is not so much with the fairness demand itself, but with what is demanded, namely vaccination. Exactly as is the case with pacifists’ exemptions from military duties, fairness would still demand that vaccine refusers make some alternative contribution to public health that could be considered equivalent to one’s contribution to herd immunity, as has recently been argued (Giubilini et al. 2017). The important point, for the purposes of the present discussion, is that individuals cannot simply escape a basic fairness demand to contribute to herd immunity; assuming for the sake of argument that for certain people for whom vaccination would entail a high psychological cost such demand of fairness does not translate into a moral obligation to vaccinate, fairness would still demand that these people make up, or compensate for their failure to vaccinate.

## Conclusion

We saw earlier that the difficulty with attributing to individuals the moral obligation to be vaccinated is due to the fact that any individual contribution to the realisation of herd immunity is negligible. We have shown that this negligibility is not enough to rule out two arguments for the existence of a moral obligation to be vaccinated, i.e. a utilitarian argument based on Parfit’s Principle of Group Beneficence and a contractualist argument.

We have also offered an additional argument for a moral obligation to contribute to herd immunity, an argument that does not require committing to problematic and not universally accepted moral theories. We have argued that there exists a duty of easy rescue—a type of duty on which most reasonable people would agree—that can be applied to collectives to ground a collective obligation to realise herd immunity. A principle of fairness in the distribution of the burdens entailed by such collective obligation allows to derive from it an individual moral obligation to be vaccinated.

Thus, we can conclude that, in spite of the negligible impact of individual vaccinations on vaccine coverage rates and on the realisation of herd immunity, there are at least three types of argument, at least one of which morally uncontroversial, that justify an individual moral obligation to be vaccinated.

Such moral obligation, in turn, strengthens the ethical justification for the imposition of coercive vaccination policies. Examples of such policies include mandatory vaccination, such as making vaccination a requirement for enrolling children in school or daycare; withholding of financial benefits for those who are not vaccinated or do not vaccinate their children; and outright compulsory vaccination. Determining which of these policies would be preferable, both from a pragmatic and from an ethical perspective, would require a separate discussion.

## Footnotes

1 We are grateful to an anonymous reviewer for raising this objection.

2 We are grateful to an anonymous reviewer for having drawn our attention to this implication.
